# Designing an instrument for police officers’ life balance and sustainable lifestyle

**DOI:** 10.1177/03080226251347181

**Published:** 2025-06-25

**Authors:** Elin Granholm Valmari, Miguel Inzunza, Ulla Nygren, Mehdi Ghazinour, Kajsa Gilenstam

**Affiliations:** 1 Occupational Therapy Unit, Department of Community Medicine and Rehabilitation, Umeå University, Umeå, Sweden; 2 Unit of Police Work, Umeå University, Umeå, Sweden; 3 School of Police Studies, Södertörn University, Södertörn, Sweden; 4 Sports Medicine Unit, Department of Community Medicine and Rehabilitation, Umeå University, Umeå, Sweden

**Keywords:** Health, health promotion, occupational therapy, patient-reported outcome measures, PROM, self-reflection, work–family balance

## Abstract

**Introduction:**

Police officers’ occupational patterns and their needs for health promotion are rarely addressed in police health research, partly due to the lack of specific instruments. This study describes the initial steps in developing an instrument that targets officers’ lifestyles and health, utilising occupational therapy and – science.

**Method:**

The study includes 48 participants and uses an instrument development methodology. First, the construct was defined through theoretical dimensions with two focus groups. Second, items were developed and tested during cognitive interviews with 28 officers. Finally, the instrument underwent content validity testing with nine occupational therapists.

**Results:**

The instrument is based on the construct of police officers’ conditions for a sustainable and healthy lifestyle. This refers to an individualised, balanced lifestyle that enables participation, choice and control, supporting healthy routines, roles and meaningful occupations across various life domains shaped by social, occupational and physical environments over time. All domains encompass aspects of working and personal life, and the balance between them.

**Conclusion:**

The instrument’s psychometric properties, representativeness and feasibility need to be continuously evaluated. Subsequently, occupational therapists and human resource personnel may use it to promote police officers’ sustainable lifestyles and life balance as an assessment and a self-reflection tool.

## Introduction and literature review

Within occupational therapy and -science, a person’s lifestyle refers to their unique occupational patterns, that is, the content and design of their lifestyle (Matuska and Christiansen, 2008). The content relates to routines, habits, rituals, roles and activity repertoires in a person’s life. The design of a person’s occupational pattern is influenced and shaped by their internal and external forces for doing. This incorporates occupational engagement and participation, occupational value and – meaning and choice and control in life. Moreover, an individual’s occupational pattern is in constant interaction with their physical, social and temporal environment, as well as with various life contexts (Granholm Valmari et al., 2024). The concept of occupational pattern also considers how occupations and roles in life are balanced while the person resides in various environments and contexts. Thus, an occupational pattern is considered sustainable if a person experiences all their needs as balanced and can organise their time and energy to meet these needs in life (Granholm Valmari et al., 2024; Matuska and Christiansen, 2008). When a person experiences life balance, the outcomes are resilience, well-being and health (Matuska and Christiansen, 2008; Moll et al., 2015). Within this perspective of balance in life lies the World Health Organization’s (WHO) goal for health promotion, namely, to enhance the physical, mental and social health and well-being of individuals (World Health Organization, 1986). Health promotion encourages people to adopt healthy lifestyles over time, thereby protecting their health and reducing future challenges to ill health (Guidotti, 2015).

Within the context of police health research, knowledge of how to promote police officers’ lifestyles is limited, with a few studies focusing on the negative consequences of shift work on sleep (Violanti et al., 2017), meal intake (Erickson et al., 2023) and problematic drinking (Ballenger et al., 2011). Research is even more scarce when lifestyle is investigated in line with occupational therapy and -science, that is, as a person’s occupational pattern. Previously, it has only been explored qualitatively (Granholm Valmari et al., 2023c). However, police officers’ work entails repeatedly engaging in confronting, risky and traumatic contexts, as well as struggling with organisational stressors, such as shift work or high job demands (Violanti et al., 2017). Moreover, police officers often carry heavy equipment, which can harm their health (Larsen et al., 2018). Police work also negatively impacts the social well-being of police officers in their private lives (Granholm Valmari et al., 2023a). For instance, having little energy left for private life needs and roles (Granholm Valmari et al., 2023c) and struggling to juggle a life partner and kids with work (Granholm Valmari et al., 2023c). Experiencing work–family conflict among police officers has been linked to stress (Duxbury et al., 2021). In summary, the mental, social and physical well-being of police officers is constantly being challenged, which negatively impacts their occupational patterns and health (Sundqvist et al., 2024; Violanti et al., 2017).

To better understand the lifestyles of police officers and the challenges they encounter in achieving a sustainable and healthy lifestyle, it is essential to adopt an occupational perspective (Granholm Valmari et al., 2023a). Thus, an in-depth examination of their occupational patterns is warranted, as it will be possible in the future to tailor specific occupational therapy intervention strategies to the needs of police officers. To gain a deeper understanding of police officers’ occupational patterns and how their health can be promoted, their lifestyle needs to be assessed and evaluated (Granholm Valmari et al., 2023a, 2023c). According to Dür et al. (2015), different aspects of occupational patterns are assessed or screened within occupational therapy. However, most focus on specific parts of an occupational pattern. They are also designed for individuals with illnesses or disabilities. Examples include balance of life, roles and activities (Dür et al., 2015), meaning (Eakman et al., 2010) and value (Eakman and Eklund, 2011). Nevertheless, instruments that specifically focus on promoting healthy occupational patterns are scarce (Dür et al., 2015). Furthermore, there is a need to assess police officers’ occupational patterns and determine how healthy occupational patterns can be promoted and sustained over time in this challenging profession (Granholm Valmari et al., 2023b).

In summary, research on police officers’ occupational patterns is limited. Moreover, suitable, psychologically robust instruments for assessing a broad range of occupational patterns and promoting health are rare within occupational science research. Furthermore, to the authors’ knowledge, no instruments exist that assess police officers’ occupational patterns. Against this theoretical backdrop, this article aims to describe the initial steps and process involved in the developmental stage of creating an instrument targeting conditions for a sustainable and healthy lifestyle among police officers.

## Method

**Figure 1.**
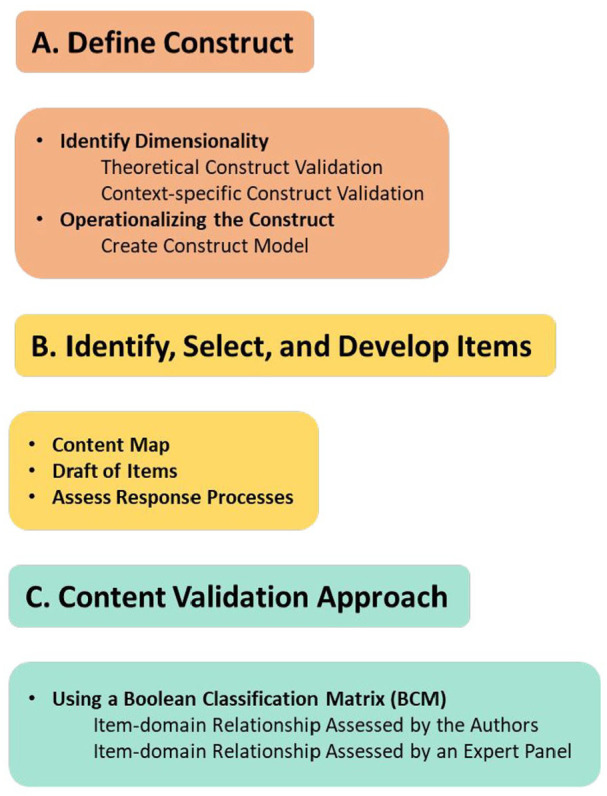
The different steps and processes during the initial developmental stages.

The study is a methodological study aimed at developing an instrument; see Figure 1 for the included steps and processes. First, the construct was defined by identifying its theoretical dimensions and operationalising it (Boateng et al., 2018). The second step was to identify, select and develop items. Items were connected to different domains of the construct, and the instrument was evaluated for relevance, representativeness and technical quality through cognitive interviews (Boateng et al., 2018). In the final step, a content validity approach was employed, utilising the initial steps outlined by Spoto et al. (2023), which consisted of evaluating the item-domain relationship. This was achieved through both author evaluation and expert assessment.

This study applied the recommendations of Flake and Fried (2020) to promote transparency in construct validation. The recommendations are comprehensive and theory-driven, ensuring the construct under investigation is assessed reliably and reflects the underlying concept. The project was approved by the Swedish Ethical Review Authority with Reg. No. 2022-03690-02, and written informed consent was obtained from all participants. Participant information and data are kept separate, and all data are encrypted and stored on local university servers in accordance with relevant data management regulations.

### Data collection and sampling procedure

Data collection in the first step included two focus groups comprising occupational therapists and experts on the health of police officers. Convenience sampling, as well as purposive sampling, was used to establish the two focus groups. In the second step, police officers participated in cognitive interviews. Convenience, purposive and snowball sampling methods were employed for cognitive interviews conducted at various stages. For instance, personal contacts within the Police Authority and social media were used. The contacts were asked to either participate or share the information if they chose not to participate. In the final step, an expert panel comprising occupational therapists was convened using convenience sampling.

The inclusion criteria for the first focus group and the expert panel were that participants should be occupational therapists with both clinical and advanced theoretical skills. The inclusion criteria for the second focus group were that the participants should be police officers, HR staff at the Police Authority and researchers with expertise in the health of police officers. The inclusion criteria for the cognitive interviews were that they should be police officers working in different frontline services. If assigned to other duties, they should consider themselves as having extensive experience in frontline services.

The focus group was conducted in person, and the cognitive interviews were conducted both in person and online. Both focus groups and cognitive interviews were recorded using a Dictaphone, and a summary was written after each session. For the expert panel, an Excel file with domains in columns and items in rows was distributed digitally to the experts. They were given 1–2 weeks to fill in the Excel file and return it to EGV.

### Procedure and data analysis

#### Define the construct

Identifying dimensions and operationalising the construct included two separate steps. First, theoretical and context-specific construct validation was undertaken to determine the dimensions of the construct. Second, the construct was operationalised (Boateng et al., 2018).

##### Identify dimensionality

To identify distinct dimensions, theoretical and context-specific construct validation was performed in separate focus groups. The method used in both focus groups was a participatory action research approach known as the diamond ranking method. This method is applied by making connections between theory and practice using visual techniques (Clark et al., 2013).

The process for both groups involved dividing participants into pairs. They ranked different theoretical dimensions of the construct according to which aspect of the construct would be most and least crucial to emphasise when used in practice for screening persons’ lifestyles. The theoretical dimensions were then ranked from most important at the top to least important at the bottom. These dimensions were life balance (including occupational balance and role balance), context and environment (both physical and social), choice and control, value and meaning, engagement and participation (including motivation), roles in life, activity repertoire (i.e. activities performed through life), routines, habits and rituals. Afterwards, a discussion among all participants in their respective groups led to a jointly formed diamond.

The theoretical construct validation was conducted with the first focus group. The focus group was planned and performed by the first author, with assistance from the last author. The participants were divided into three groups to identify and rate different dimensions of the theoretical construct of occupational patterns. They rated which dimensions would be most important, regardless of target group, that is, not explicitly focusing on police officers. Furthermore, they were provided with instructions that we were specifically interested in, namely, ‘conditions of a person’s lifestyle regarding what is sustainable and healthy’.

Context-specific construct validation was performed with the second focus group. The focus group was planned and led by the first author, with assistance from the third author. The participants worked in pairs and were provided with the same theoretical dimensions of the occupational patterns construct as those presented in the first focus group. This group also received information regarding what each dimension specifically includes for police officers. The task was to rank the different dimensions by what would be most important to focus on if the instrument were explicitly designed to target conditions for a sustainable and healthy lifestyle of police officers.

The construct’s theoretical content validity was assessed among the raters using Krippendorff’s alpha for focus group one and weighted kappa for focus group two. The calculations were conducted in SPSS (IBM Corp., 2024). The intention was to test the theoretical assumptions and the concept itself before constructing items. Both content validity measures produce a value between 0 and 1, where a higher value indicates greater agreement among the raters.

##### Operationalising the construct

The first author developed a construct model and definition through a synthesis of data from the focus groups, along with two previously conducted reviews. MAXQDA (VERBI Software, 2025) and Microsoft Word (Microsoft Corporation, 2018a) were used to synthesise the data. The first review focuses on the contexts and environments affecting police officers’ lives and health (Granholm Valmari et al., 2023b), and the second describes police officers’ balance in life and its effect on physical, social and mental health (Granholm Valmari et al., 2023a). The construct was defined in relation to its intended target within a police-specific context, and a construct model was developed, along with the definition of the construct and descriptions of the model’s included domains. The other authors provided feedback on the model.

#### Identify, select and develop items

Three steps were used to identify, select and develop items. First, a content map was created, and then a set of initial items was assembled. Lastly, response processes were assessed.

Using the dimensions from the construct model in step A, the first author created a content map, connecting the items to every domain in the model. Both inductive and deductive methods (Boateng et al., 2018) were employed to generate indicators. Microsoft Word and Microsoft PowerPoint were used (Microsoft Corporation, 2018b). A previously conducted study with in-depth interviews (Granholm Valmari et al., 2023c, 2023d) (inductive method), together with two previously conducted reviews (deductive methods; Granholm Valmari et al., 2023a, 2023b), were utilised. Lastly, other instruments that measure occupational patterns and lifestyles’ content, process and balance were investigated. This added to the already generated pool of items and added indicators that tap into the domain of the construct. Hence, an initial draft with items was created, and the third and final authors provided linguistic feedback on the first set of items.

Response process validity was assessed using cognitive interviewing techniques with police officers (Hak et al., 2008). Cognitive interviews are used to test items that make up the domain of representativeness of actual experience (Hak et al., 2008).

The first author conducted the cognitive interviews using the three-step test-interview (TSTI) method (Hak et al., 2008). TSTI is an observational instrument for pretesting self-completion instruments. It involves observing actual instances of interaction between the respondents and the instrument (the response processes). The first step involved a concurrent think-aloud session aimed at collecting observational data, followed by focused interviews to address gaps in the observed data, and a semi-structured interview to elicit experiences and opinions (Hak et al., 2008). The questions for the semi-structured interview were inspired by Slade et al. (1999), who suggested that the psychometric properties of feasibility instruments include brevity, simplicity, relevance, acceptability, availability and value (Slade et al., 1999).

The wording of items, instructions and response format were tested. After every turn, the feedback from the participants was summarised in Microsoft Word (Microsoft Corporation, 2018b). The summaries and revised instruments were sent to the other authors for comments between clusters. Then, the instrument was revised by the first author, and a subsequent cluster of participants tested the revised instrument.

The ecological validity of the instrument (Holleman et al., 2020) was important during the response process. The stimuli, in this case, the items, must be precisely allocated in the correct order to elicit answers. Furthermore, as the instrument was intended to be an online tool, an effort was made to visually enhance its layout, specifically in the research context, which was designed for future use. The purpose was to transition from artificial to natural and from simple to more complex in a systematic approach, already during the development of the instrument. Furthermore, describing the theory of how specific environmental contexts are related to various forms of behavioural functioning (Holleman et al., 2020). In this case, how the context of using the instrument for self-reflection might prompt behavioural change by applying the instrument in a real-world setting.

In summary, before entering the content validity stage, we focused on ensuring relevance, comprehensibility and comprehensiveness – ensuring that all relevant items were included, explicit and covered all aspects of the construct.

#### Content validation

A version of the newly developed ‘Formal Content Validity Analysis’ (FCVA) by Spoto et al. (2023) was employed to assess content validity, including the initial steps of the method, specifically the ‘author-item domain relationship’ and the ‘expert-item domain relationship’. It was deemed the most rigorous method as it includes both author and expert evaluations, which allows for the detection of four critical situations that should be avoided for the sake of good content validity and high assessment instrument efficiency, that is, empty items, completely redundant items, lack of exhaustiveness or completely redundant elements (Spoto et al., 2023).

The author’s evaluation of the relationships between items and domains includes an initial analysis of a Boolean Classification Matrix (BCM). Since the construct of ‘conditions for a sustainable and healthy lifestyle of police officers’ is a complex construct, higher-order domains were rated instead of elements, as the original method indicates. Microsoft Excel was used (Microsoft Corporation, 2018a). A BCM was created by the first author, classifying items into different domains of the construct. A ‘1’ (affirmative) was noted for the specific items belonging to each domain. In the second step of the FCVA, according to Spoto et al. (2023), the relationship between items and elements was evaluated by an expert panel comprising nine occupational therapists. The evaluation of agreement between experts for the entire set of items provides an overall picture of the consistency of the items–domain relationship. Each expert individually assesses whether a particular item belongs to a specific domain and assigns a ‘1’ (indicating an affirmative response) within the respective domain of the construct in their BCM (Spoto et al., 2023).

## Results

The study, conducted in 2022 and 2023, presents an initial development process for an instrument with potential applications for both screening and self-reflection regarding the conditions for a sustainable and healthy lifestyle among police officers. See Table 1 for the results of those who participated in the study. Forty-eight participants, including men and women, were enrolled in various parts: 11 in focus groups, 28 in cognitive interviews comprising 8 separate clusters and 9 in the expert panel.

**Table 1.** The participants during focus groups, different clusters of cognitive interviewing and expert panel.

**Focus Group 1**	**The focus group comprised seven female occupational therapists. They are all practically and theoretically skilled in occupational science and occupational therapy and should be considered experts on the construct under study. Furthermore, four of them are also doing research in occupational science and** – **therapy.**	
**Focus Group 2**	The focus group contained four experts, two women and two men. They were chosen for their specific skills related to police officers’ lifestyle and health, including various competencies. One of them was a police health researcher, two were HR staff working for the Swedish Police Authority, and one person was responsible for scheduling police officers’ shifts.	
**Cognitive** i**nterviews**	Cluster 1	Two men (one police officer doing patrol service and one dog handler)
	Cluster 2	Two men (two police officers previously doing patrol service but now doing different assignments for the Police Authority)
	Cluster 3	Three men (one police officer doing patrol service, one police officer working undercover and one police officer previously doing patrol service but now doing other police work)
	Cluster 4	Three men and one woman (one former Swedish counter-terror intervention officer now doing other police work, and three police officers doing patrol service)
	Cluster 5	Four men and one woman (one police officer previously doing patrol service but now doing other police work, four police officers doing patrol service)
	Cluster 6	Two men and two women (two police officers doing patrol service and two police officers previously doing patrol service but now doing other work for the Police Authority)
	Cluster 7	Six men (all of them police officers doing patrol service)
	Cluster 8	One man and one woman (both police officers doing patrol service)
**Expert** panel	Nine occupational therapists (six women and two men) familiar with the concept of occupational patterns were asked to validate the instrument. Five of these persons were the same as in focus group 1. All of them possess both clinical and theoretical skills in working with individuals’ occupational patterns.	

### Operationalising the construct: Creating a construct model

Seven occupational therapists, who were regarded as experts on the theoretical construct, participated in the first focus group. Four experts on the lifestyles and health of police officers were included in the second focus group. Six participants were initially enrolled in this stage; however, two were unable to participate due to sudden unavailability.

First, the findings from both focus groups were distilled to identify the dimensions of the construct ‘conditions for a sustainable and healthy lifestyle’, creating a construct model. Although the focus groups had slightly different foci, they shared some mutual areas on what should be covered in a screening for a sustainable lifestyle and health. The inter-rater reliability of the theoretical assumptions for the three groups in focus group 1 was 0.51, indicating disagreement regarding the importance of the different domains within the construct. They were also urged to forge a mutual diamond but were unable to do so with two of the domains. Regardless, value and meaning were found to be the most important to measure.

The second focus group tended to agree even less. Inter-rater reliability was 0.31. When forging a mutual diamond between the two groups and discussing the dimensions among each other, they were more inclined to agree on the importance of the different domains. Hence, a balanced lifestyle was considered the most important, serving as the foundation of the entire construction. A quote from focus group two concluded, ‘. . . having balance in life as well as choice and control, then everything else resolves itself’. In line with the first focus group, the second focus group regarded choice and control as important, as well as value and meaning in life. Routines and habits were rated high for both focus groups, although not highest for either group. Being motivated and finding engagement in life was found to be essential for both groups, although they ranked it slightly differently. Managing time was another domain found to be crucial when screening for a sustainable lifestyle.

The construct model, which includes a construct definition, considers the inter-rater reliability between the different groups in the two focus groups. The following definition was constructed, at this point including five domains, that is: ‘Health and safety in all contexts and environments in life’, ‘Managing and Organising time in everyday life’, ‘An everyday life including value and meaning’, ‘An everyday life including engagement and development’, ‘Exercising choice and control to balance activities, roles and energy depots’ (Table 2), indicating what the conditions are for a sustainable and healthy lifestyle for police officers:

An individualised, balanced lifestyle is one where a police officer’s occupational patterns allow for participation, choice, and control, supporting healthy roles and occupations in life. It is a lifestyle where the police officer feels empowered within their environments (e.g., social, occupational, and physical) and contexts in life (e.g., working in a male-dominated organisation or having a family). The transaction between contexts and environments, as well as the roles of police officers, encompasses their daily routines, habits, and rituals. These everyday activities and roles in life need to be congruent with the police officers’ values over time and experienced as balanced to meet essential life needs. Overall, the police officer, their environment, context, and their daily activities are interdependent and inseparable.

**Table 2.** Example of BCM and the different ratings of all raters on a few example items.

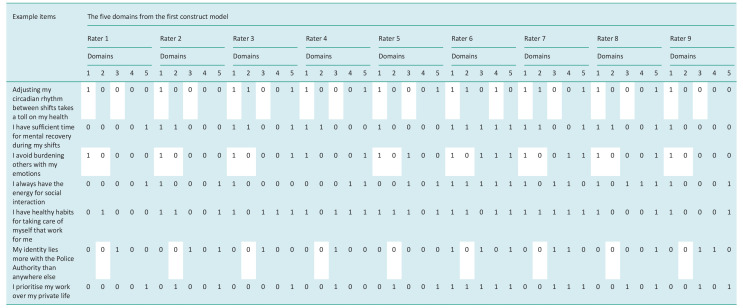

The first five domains created for the construct model, that is.; ‘Health and safety in all contexts and environments in life’, ‘Managing and Organising time in everyday life’, ‘An everyday life including value and meaning’, ‘An everyday life including engagement and development’, ‘Exercising choice and control to balance activities, roles and energy depots’. Similar ratings between raters are coloured in white, where a ‘1’ indicates in which domain the rater considers the item to belong. Contact the first author; more example items can be provided upon reasonable request.

### Identifying, selecting and developing items

The TSTI was used in eight clusters involving two to six persons. The third cluster was the one where the participants filled in the instrument online for the first time. For practical reasons, the cluster sizes were determined based on the number of participants enrolled in the study and the need for amendments between the clusters.

The instrument is structured to be context-sensitive and experienced as relevant. At the beginning of every section of the instrument, there are a few questions regarding, for example, social status, age, gender and frequency of activities in life, which serves as an introduction to each section. Furthermore, the scales in the instrument differ, as it includes both questions and statements to consider. For the questions, there are three to four options to consider regarding frequency. Some questions have yes/no options. For the statements, a bipolarity scale was chosen due to its capacity to measure opinions better. The scale has seven steps, ranging from ‘absolutely disagree’ (−3) to ‘totally agree’ (+3), with a 0 at the middle indicating ‘neither/nor’. There is also one last option indicating ‘Not relevant for me’. We wanted to avoid people choosing the middle option for rating the different statements in the instrument, as this has been regarded as a possible problem with bipolarity scales. Hence, this alternative step was selected to strengthen the possibility of choosing the correct answer, as this approach has proven to be valid, thereby reducing the uncertainty for the test-taker. Interpretations of the scores have not yet been considered in this stage of the process and will be developed in the next phase.

During the cognitive interviews, certain items were generally well-received, with police officers expressing few objections. These items were straightforward and required minimal cognitive effort to understand. For instance, which type of shift work affects their lifestyle and health the most negatively, how much they exercise in their private lives, or what kinds of leisure activities they participate in. In contrast, some items were more abstract and complex, making them difficult for some participants to grasp. For example, statements related to whether they had activities in life that were mentally draining or what part of their life, such as work or family life, was found to be more engaging. Certain items had multiple interpretations, prompting discussions to clarify their intended meanings for future testing in new clusters. Additionally, neutral items lacked a clear purpose, raising debates on whether they should be reworded to elicit more informative responses, either positively or negatively. Items deemed irrelevant to the construct, target population or context were excluded during the cognitive interviews.

The instrument also included several items regarded as highly sensitive, that is, items related to sex life, and potentially stigmatising items, that is, items concerning mental health and suicide ideation. This led some respondents to express concerns about others’ willingness or ability to answer truthfully without assurances of confidentiality. This raised the risk of nonresponses or dishonest answers. Consequently, early in the cognitive interviews, it became clear that encouraging honest responses to uncomfortable questions was crucial to maintaining construct validity and reliability, ensuring the instrument measures accurately and remains relevant. A significant effort was made to specifically ask about these items, to remove or rephrase them, and to reassign them to suit theories of ecological validity. Hence, by the later stages of the interviews, most police officers engaged with the items, including sensitive ones, as efforts had been made to address their experiences and behavioural responses to the instrument. They also expressed their interest in using the instrument for self-reflection.

### Content validity evaluation of the instrument

Since the instrument evolved from theoretical frameworks within occupational science and -therapy, nine occupational therapists were used as experts. Hence, after the author evaluates the item-domain relationships, the experts fill in the same BCM. The content validation process revealed ambiguities in the construct definition and its specific dimensions, highlighting the need for more precise and distinct domain definitions. See an example of these ambiguities in Table 2, which illustrates how the ratings may differ between raters.

**Figure 2.**
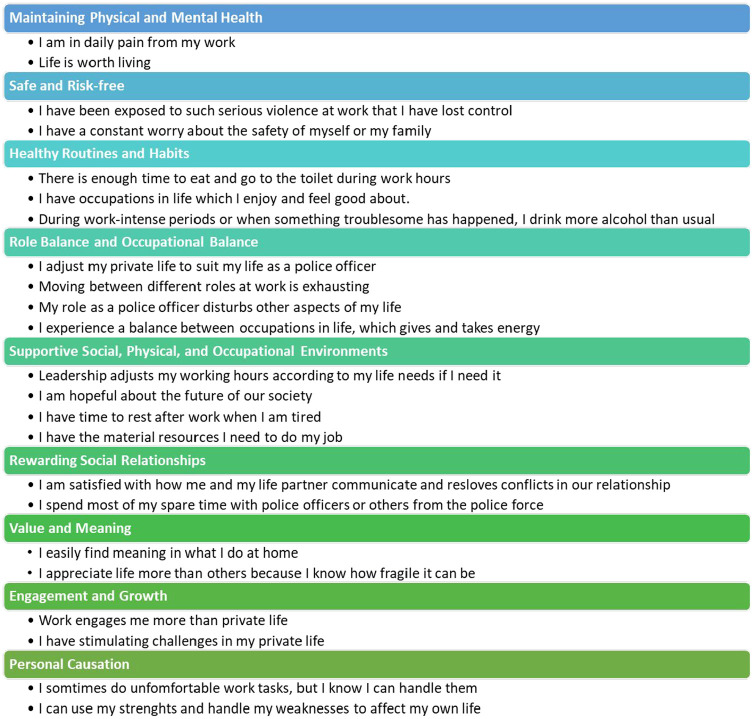
Domains included in the construct model, including sample items. Under each domain are sample statements for each dimension in the instrument.

In addition to the BCM ratings, some participants provided qualitative feedback. Together with the item–domain relationship assessed by the authors, the five domains in the previously created construct model were expanded to nine domains. Three domains were each split into two, and an additional domain relating to personal causation was added. A final construct model was created, which now includes nine domains (see Figure 2). All domains encompass aspects of both working life and private life, or how these areas intersect, enrich each other, or strike a balance between them. Further steps are necessary for content validity testing of these new domains before proceeding with the additional steps outlined in the FCVA method by Spoto et al. (2023).

## Discussion

This study aimed to describe the steps and processes involved in the initial development stages of an instrument designed to target conditions for a sustainable and healthy lifestyle among police officers. By defining the construct and identifying, selecting and developing items, we now have a theoretical model that includes items representing each dimension. This aligns with the transparency in instrument development, as Flake and Fried (2020) noted, which has influenced our study’s design and presentation. Following Holleman et al. (2020), we also intend to discuss and describe the theories that relate specific environmental contexts to various forms of behavioural functioning, as the instrument also shows potential for self-reflection. That is, how the context of using the instrument for self-reflection might have provoked behavioural change when used in a real-world context. Thus, we aim to provide a theoretical explanation and discuss how the instrument can be utilised for future purposes, both in screening the construct itself and as a self-reflection tool in future interventions.

First, the instrument can be used to screen for conditions that promote a sustainable and healthy lifestyle among police officers and possibly measure individual changes within these conditions. If the instrument proves valid for screening, it could contribute to the development of new research within the field of police officers’ health, providing insights into the lifestyle and life balance of police officers. Thus, the instrument could offer a more comprehensive approach to gathering data on police officers’ lifestyles and health than current instruments. However, rigorous psychometric construct validation is needed to determine if the instrument possesses the necessary properties for screening the construct. As a first step, content validation should be conducted again with the new domains, following all steps as outlined by Spoto et al. (2023). Moreover, reliability testing is necessary (Furr, 2022).

Second, there is a possibility of using the instrument as a self-reflection tool, as it was found to have relevance and fulfil meaning for the police officers using it. According to Grant et al. (2002), effective self-reflection comprises three key elements: first, an awareness of the need for self-reflection; second, a willingness to engage in self-reflection; and lastly, the ability or insight required for self-reflection (Grant et al., 2002). The instrument could potentially target all of these aspects as the participants became aware of their needs, engaged in self-reflection and expressed a need for more self-reflection. According to O’Brien and Kielhofner (2017), individuals think and feel about their actions and performance in life; that is, they engage in self-reflection, which also lays the foundation for their future. Hence, to achieve sustainability in life, we need to feel, think and act/behave accordingly (O’Brien and Kielhofner, 2017). Therefore, focusing on everyday life and various roles, habits and occupations can provide a solid foundation for promoting the lifestyles and health of police officers.

According to Arble et al. (2018), there is also a need for police-specific interventions to promote the health of officers, enabling them to adopt healthier habits and improve their health outcomes. Police officers are prone to certain lifestyle factors that may turn into ill health, such as high alcohol consumption, influenced by factors such as workplace drinking culture, trauma and stress (Ballenger et al., 2011). Previous research has concluded that the health promotion interventions available for police officers internationally have been suboptimal (Zimmerman, 2012), and none of them are tailored to the Swedish context. Hence, the instrument may provide a tailored intervention tool designed to cater specifically to the needs of police officers, thereby promoting their health. Moreover, it has also been argued that achieving balance (i.e. experiencing occupational, role or life balance) within one’s occupational pattern is linked to lower perceived stress, higher subjective well-being, better-perceived health and improved quality of life (Moll et al., 2015). As police officers work in challenging contexts (Granholm Valmari et al., 2023b), also affecting their lifestyle and health (Granholm Valmari et al., 2023a), the need for achieving balance in life is crucial to sustaining both at work and in personal life (Granholm Valmari et al., 2023c). In turn, promoting resilience and well-being plays a significant role in maintaining health and preventing illness (Matuska and Christiansen, 2008).

Third, this study has demonstrated the potential relevance of the instrument during cognitive interviewing, providing value to participants in their reflections on life, even if some items were found to be stigmatising. Police officers often face difficulties in seeking support from healthcare services when needed, partly due to stigmatisation, which presents a significant problem (Berg et al., 2006). However, this study suggests that a context-sensitive instrument may facilitate self-reflection, even on stigmatised topics. For instance, the instrument has been tailored to both a police-specific and a male-dominated context. According to Robertson et al. (2018), health promotion interventions should also be developed and delivered in gender-sensitive ways for both men and women when designing interventions in male-dominated contexts. For example, for men, this entails focusing on activities in life to initiate engagement in health promotion activities, which is a good way to facilitate health behaviour in a non-threatening manner (Robertson et al., 2018). According to Seidler et al. (2016), tailoring standard clinical interventions for men may increase the uptake, adherence and efficacy of treatment. For example, this can be achieved by offering more problem-solving treatment, promoting and sustaining behavioural changes in the long run (Seidler et al., 2016).

The instrument could become a valuable self-reflection tool for police officers to assess their sustainability in terms of lifestyle and health, allowing them to discuss sensitive topics and gain insights into what constitutes a sustainable lifestyle on a personal level. According to Sharp et al. (2022), it is essential to focus on men’s skill-building within a non-stigmatising environment when promoting men’s health (Sharp et al., 2022). This is also in line with occupational therapy, where a person is constantly in transaction with what they do and the environment and context in which they are (O’Brien and Kielhofner, 2017). As a result, environments are regarded as crucial to an individual’s change process and are inextricably linked to a person’s occupational performance (Fisher et al., 2017).

### Methodological discussion

This study has several limitations. One limitation is not utilising qualitative content validation beyond the feedback provided on the various items of the instrument. Hence, combining quantitative indices of content validity with qualitative feedback from raters, as Haynes et al. (1995) suggested, would have been ideal.

Second, both lay experts and content experts could have been included in the study, as suggested by Boateng et al. (2018). Thus, the expert panel may comprise different experts based on their expertise in the topic to be studied (Rubio et al., 2003). If a concept is complex, experts from several disciplines may also be used. However, due to the instrument evolving from theoretical models and research within occupational science, it was deemed most suitable for occupational therapists to evaluate content validity. This should be considered a strength of the study, as the researchers are most familiar with the construct under study.

Third, we applied the FCVA developed by Spoto et al. (2023) to assess content validity. Content validity is typically established by relying on experts familiar with the construct to evaluate the test content (Furr, 2022). The number of experts is recommended to be between 3 and 20 (Rubio et al., 2003), and this study used nine participants. If an instrument is developed from a theoretical framework, it is best reviewed by experts in the theory or concept (Davis, 1992). FCVA offers a theory-driven framework that integrates both author and expert evaluations to assess how effectively each item represents the intended construct. While the method is still emerging in the literature, with only one known empirical application to date (Bacchi, 2025), its formal structure and integration of inter-rater agreement, including both authors and experts, may offer advantages over traditional approaches. Hence, this study also contributes to the growing application of FCVA in applied research. Regardless, the entire FCVA was not possible to conduct, as we experienced issues with the domain. Hence, the content validity process needs to be repeated before proceeding to the other steps in the FCVA and the reliability testing. It is regarded as impossible to measure reliability if content validity doesn’t exist (Terwee et al., 2018).

Additional considerations were made regarding the scaling of the instrument, where the participants were highly involved in the development of the scale steps. This should be considered a strength of the study.

## Conclusion and contribution to the field

In summary, our study outlines the initial development process of an instrument with potential use for both screening and self-reflection regarding conditions for a sustainable and healthy lifestyle among police officers. Its psychometric properties still need to be evaluated for suitability for screening these conditions. Such an instrument could, for instance, assist healthcare professionals and human resource personnel in identifying an appropriate individualised mix for a sustainable and healthy lifestyle for each police officer. The instrument could also be used as a self-reflection tool, raising awareness of one’s lifestyle. Nevertheless, the instrument needs to be tested for representativeness and feasibility in the contexts where it is intended to be applied, and a more extensive feasibility study should be considered.

**Key findings**

- A new instrument has been developed to assess sustainable lifestyle and life balance among police officers.
- Future validation efforts will require more explicit construct definitions.
- The instrument also shows promise for promoting self-reflection.

**What the study has added**

This study presented a construct model for evaluating sustainable and healthy lifestyles among police officers, identifying key areas for refinement in item clarity and domain specificity to inform future instrument development. The instrument shows potential for use as self-reflection.
